# Factors associated with sexual dysfunction in Jordanian women and their sexual attitudes

**DOI:** 10.4103/0256-4947.55312

**Published:** 2009

**Authors:** Ruba M. Abu Ali, Rabaa M. Al Hajeri, Yousef S. Khader, Kamel M. Ajlouni

**Affiliations:** From the National Center for Diabetes, Endocrinology and Genetics, Amman, Jordan

## Abstract

**BACKGROUND:**

Female sexual dysfunction (FSD) is defined as disorders of libido, arousal, and orgasm, as well as sexual pain, that leads to personal distress or interpersonal difficulties. Social aspects of FSD have been understudied. The aim of this study was to explore the social aspects of FSD and sexual attitudes of Jordanian women.

**SUBJECTS AND METHODS:**

Six hundred thirteen married females were studied between October 2006 and August 2007 at the National Center for Diabetes, Endocrinology and Genetics (NCDEG), Amman, Jordan. Females were interviewed using a special questionnaire that was suitable to our culture and added to the Arabic translation of the Female Sexual Function Index (FSFI) Questionnaire.

**RESULTS:**

Older age was associated with a decreased total FSD index and its domain scores. Women with obesity were more likely to have impaired arousability and impaired capability of reaching orgasm. About 58.5% of women reported that they prepared themselves if they had sexual desire and 68.2% reported wearing special attire for this purpose. Only 37.2% of women could ask their husband for a special excitement.

**CONCLUSIONS:**

FSD is prevalent in Jordan. Its social aspects are understudied and need more research in the future.

Sexuality is an important part of health, quality of life and general well being.^1^ Sexual dysfunction impacts significantly on a women's self-esteem and quality of life and causes emotional distress, leading to relationship problems.^2^,^3^ Previous studies showed that the prevalence rates of sexual dysfunction among all women ranged between 25% and 63%.^4^–^6^ In postmenopausal women, the prevalence of sexual dysfunction varied from 68% to 86.5%.^7^,^8^ Relatively little research has focused on the social factors that are associated with sexual dysfunction and the sexual attitudes of women.

Islam is a religion that esteems women and places importance on the marital relationship and foreplay in achieving satisfaction. Aspects like preparation for coitus and proper knowledge about marital relationships before getting married are integral parts of Islamic behavior.^9^ Data on sexual behavior and sexuality among women in Islamic countries are sparse. This study aimed to address the social aspects of sexuality among Jordanian women and determine factors associated with sexual dysfunction.

## SUBJECTS AND METHODS

A systematic random sample of 613 married women was selected from all females who attended the National Center for Diabetes, Endocrinology and Genetics (NCDEG) in Jordan between October 2006 and August 2007. In systematic random sampling, first a number within the sampling interval was chosen. We chose a random number between 1 and 10 using random number tables. Then every 10th woman following the first number chosen was selected each day for the whole study period. Divorced, widowed or seriously ill women were excluded from the study. This study was approved by the NCDEG ethics committee. Participants were informed about the objective of the study, and asked to read carefully and sign a consent form, which was also approved by the ethics committee. All women agreed to participate in this study and signed the consent form. A face-to-face interview was held by a female researcher while assuring privacy and confidentiality to collect data. Demographic and clinical data were recorded. Women were asked to answer selected culture-sensitive questions (Appendix 1) to assess their behaviors and attitudes toward sexual functioning. In addition, the structured interviews were based on the 9-items of the Female Sexual Function Index (FSFI) Questionnaire,^10^ which was translated to Arabic, and tested for validity and reproducibility. Scores of the six domains, which included desire, arousal, lubrication, orgasm, satisfaction and pain, were added to obtain the full-scale score. For individual domain scores, scores of the individual items that comprise the domain were multiplied by the domain factors, where higher scores indicate less dysfunction.^10^

Weight and height were measured while the subject was wearing light clothes and not wearing shoes. Weight was taken to the nearest 0.5 kilogram and height was taken to the nearest 0.5 centimeter. Body mass index (BMI) was calculated as weight in kilograms divided by height in meters squared. Females were categorized as being of normal weight (BMI<25 kg/m^2^), overweight (BMI=Age and body mass index 25-29.9 kg/m^2^), or obese (BMI≥30 kg/m^2^).

The Statistical Package for Social Sciences software (SPSS, version 15, Chicago. Inc) was used for data processing and data analysis. Characteristics of subject variables were described using frequency distribution for categorical variables and mean and standard deviation for continuous variables. The multivariate analysis of the association between FSFI domains and independent variables was conducted using the general linear model multivariate procedure. This procedure provided regression analysis and analysis of variance for multiple dependent variables (FSFI domains) by explanatory variables and covariates. A *P* value of less than .05 was considered statistically significant.

## RESULTS

About half (47.8%) of the 613 women were younger than 50 years, 35.2% were between 50 and 59 years, and 17% were 60 years or older. Only 10.2% of women had a BMI<25 kg/m^2^, 31.9% were overweight and 57.9% were obese. In the multivariate analysis, older age was associated with decreased total FSDI scores and its domains scores (Table 1). Compared to those with normal BMI, obese subjects were more likely to have impaired arousability and impaired capability of reaching orgasm.

**Table 1:** Multivariate analysis of factors associated with total female sexual dysfunction index by domain scores.

Variable	Desire	Arousal	Lubrication	Orgasm	Satisfaction	Pain	Total
**Age (years)**							
<50	3.6 (1.4)	3.6 (1.6)	4.3 (1.6)	4.1 (1.7)	4.2 (1.5)	2.6 (1.2)	22.7 (7.4)
50-59	3.0 (1.5)	3.0 (1.7)	3.6 (2.0)	3.5 (2.0)	4.0 (1.8)	2.4 (1.4)	19.8 (8.8)
>60	2.5 (1.4)	2.5 (1.7)	2.9 (2.1)	2.9 (2.1)	3.6 (1.8)	1.9 (1.2)	16.6 (8.9)
*P*value	.0001	.0001	.0001	.0001	.087	.0001	.0001
**Body mass index**							
Normal	3.5 (1.5)	3.4 (1.6)	4.1 (1.7)	4.0 (1.7)	4.1 (1.6)	2.7 (1.2)	22.1 (7.6)
Overweight	3.3 (1.5)	3.4 (1.7)	3.9 (1.9)	4.0 (1.9)	3.9 (1.7)	2.2 (1.1)	21.2 (8.7)
Obesity	3.1 (1.5)	3.1 (1.7)	3.7 (1.9)	3.5 (1.9)	4.1 (1.7)	2.5 (1.3)	20.3 (8.4)
*P*value	0.055	0.036	0.165	0.005	0.723	0.242	

About 41.1% of women considered their kids the most important person in their lives, while only 33.8% considered their husbands as the most important. When they were asked to identify factors that might hinder their sexual functioning, 23% identified lack of water, 24.1% identified lack of privacy and 36.4% identified kids being awake at night as the most important factors. About 51.3% of women reported that they keep themselves neat on a daily basis during time spent at home and only 54.3% keep themselves neat for their husbands return from work. This is compared to 61.7% of women who reported that they keep themselves neat for a women's gathering. About 58.5% of women reported that they prepare themselves if they have sexual desire and 68.2% reported wearing special attire for this purpose. About 67.1% of females reported that they keep themselves ready if they feel that their husbands have sexual desire. As for Friday (weekend) night, it was considered a special night for 28.4% of women, where 44.8% of them wear special clothes, 40.1% put on makeup and 46.3% use a special perfume. Only 37.2% of women could ask their husband for a special excitement and 36.8 % of them would ask for a specific sexual position. About 84.6% reported being kissed before coitus, 83.4% during and 51.9% after finishing intercourse.

## DISCUSSION

Sexual attitudes differ between different societies, among different eras, between different individuals and even in the same person when times and circumstances change.^3^ Sexual intercourse is not the only component of a marital relationship.^3^ It is emotional sharing and mutual sentiments that culminate in intercourse, preceded by sufficient foreplay that leads to satisfaction and pleasure in both the man and woman.^3^ The importance of foreplay is underscored and recommended strongly in Islam and it is mentioned in the holy Quran and the Hadith (Table 2). For foreplay to take place, the elements of preparing oneself via cleanliness and having a pleasant odor should be present. This applies to both husband and wife.^3^ For the wife to put on special clothes, wear perfume and makeup-if desired-are suitable anticipatory actions that are encouraged because they pave the way for a successful encounter.^3^ There are no restrictions as far as kissing and talking during coitus. In fact that is stressed in Islam but, unfortunately, ignored in some cultures because of misconceptions or traditions. The same applies for postcoital kissing and cuddling. There are no borders between a husband and wife as far as the position or kind of excitement desired during intercourse,^3^ a fact that is also shadowed by traditions and misconceptions.

**Table 2:** Passages from the holy Quran.

*“Your wives are as a tilth unto you so approach your tilth when or how ye will; but do some good act for your souls beforehand and fear Allah, and know that ye are to meet him (in the thereafter), and give (these) good tidings to those who believe* (The holy Quran, Al Baqara, verse 223).”
*“Permitted to you on the night of the fasts, is the approach to your wives. They are your garments and ye are their garments. Allah knoweth what ye used to do secretly among yourselves, but he turned to you and he forgave you, so now associate with them, and seek what Allah hath ordained for you, and eat and drink until the white thread of dawn appears to you distinct from its black thread; then complete your fast till the night appears; but do not associate with your wives while ye are in retreat in the mosques. These are limits (set by) Allah; approach not night thereto. Thus doth Allah make clear his signs to men: that they may learn self- restraint* (The holy Quran, Al Baqara, verse 187).”

In our study, the issue of the mutual respect and treatment between man and woman had an impact on FSD, which goes back to the core of Islam emphasizing the essentiality of treating women well and the fact that the emotional and psychological aspects of marital relations are as important as the physiological ones.^3^ Keeping themselves neat and at times tempting only for the husband, as required in Islam, was not the trend of the women in our study, who kept themselves neat for outside visits and women's gatherings more than for the husband at home. Nonetheless, not few were those women who were aware of Islamic teachings of preparing oneself for the husband if they or their men had the desire for marital relations. Kissing and cuddling were integrated into the marital relations, before and during coitus to a larger extent than after, which shows an awareness of Islamic behaviour. This is in accord with the Hadith which stressed the kiss as the best messenger in foreplay. However, this did not apply to aspects of position and special excitements during coitus, which few women could ask for.

Our findings that age and BMI have a negative impact on female sexual function are in accord with other studies^6^,^7^ but in contrast to the study of Enzlin and associates, in which no association was found between age, BMI and FSD.^8^

In our study, females holding a diploma degree had FSD more than others. Higher education level was associated with more FSD in the study of El Nashar et al.^4^ The higher incidence of sexual dysfunction in better educated individuals is surprising given that they are healthier and have lifestyles that are less physically stressful and emotionally demanding. However, a higher educational level may also be associated with an increase in ability to freely express their dissatisfaction.^4^ This finding agrees with those of van Gleen et al,^9^ who showed a positive correlation between level of education and sexual dysfunction. In our study, female employment had a significant impact on FSD, in contrast to the findings of El Nashar et al.^3^ Privacy was an issue in FSD in our study as well as in the study of El Nashar et al.^3^ Nonetheless, socioeconomic status did not have a significant impact on FSD, which is in accord with the findings of El Nashar et al,^4^ but in contrast to the findings of Laumann et al.^10^

The records of the Jordanian Islamic courts have an estimate of 13% for polygamous marriages (for the years 2002 to 2006) in comparison to 10.7% in our study, bearing in mind that the court records are more accurate because in reality some women do not know that their husbands are married to other women. Nonetheless, and as far as we know, there were until now no studies that addressed issues of polygamy and FSD, and other social aspects of the issue, such as the effect of the mode of marriage on FSD and female preparation for coitus. The frequency of polygamy and related factors needs further study. One of the shortcomings of the study is that only women were interviewed and some issues require the opinion of the husband. Studies involving couples would be good in the future.

In conclusion, FSD is prevalent in Jordanian females and the sexual attitudes of these women need to be explored in more detail in future research.

## Appendix 1

Special questionnaire used to assess the social aspects of female sexual dysfunction.

**Table:**

If you were to choose the most important person in your life in terms of priority, it would be: yourself, your husband, your parents (mother, father, both), your kids (males, females, both), others (brothers, sisters, others). Is your husband married to another woman? Does the lack of water (hot water specifically) constitute a problem? Is lack of privacy a problem? Does the presence of kids and their number (especially if they stay awake late at night) constitute a problem? Did you have information about marital life prior to getting married? If yes, did you get information from: mother, female friends, books, magazines, films? Was your marriage traditional or by love? If traditional, was it to a non-relative? First-degree cousin? Second-degree cousin? Do you remember your weight at marriage? When did your weight start to increase? After marriage? After your first kid? Do you keep yourself neat on daily basis while at home? Do you keep yourself neat if your husband comes and tells you he's coming home? Do you prepare yourself if your husband is taking you out? Do you prepare yourself if you're going out for a women's meeting? Do you prepare yourself if you had desire for marital relation? Do you wear special clothes if you had a desire for sex? Do you prepare yourself if you feel your husband wants to have sex? Do you wear special clothes to seduce your husband if you had the desire for a marital relation? Is Friday night considered a special day to you? (Do you wear special clothes then? Special makeup? Special perfume?) Was your husband nicest in his twenties, thirties, forties, fifties, sixties, or he did not change all through? Can you ask your husband for any special excitement during sex? Can you ask your husband for a special position during coitus? Does your husband kiss you in general? If yes, as he comes in home? as he comes from travel? before coitus? During or after coitus?
